# Evaluation of methods to assess the quality of cryopreserved *Solanaceae* pollen

**DOI:** 10.1038/s41598-023-34158-z

**Published:** 2023-05-05

**Authors:** Nathalia S. M. Langedijk, Silvan Kaufmann, Ellen Vos, Tanja Ottiger

**Affiliations:** 1 Enza Zaden Seed Operations B.V., Haling 1E, 1602 DB Enkhuizen, The Netherlands; 2 Amphasys AG, Technopark Lucerne, 6039 Root D4, Switzerland

**Keywords:** Pollen, Pollen tube

## Abstract

*Solanaceae* pollen cryopreservation is a common practice in the hybrid seed production industry worldwide, enabling effective hybridization across geographical and seasonal limitations. As pollination with low quality pollen can result in significant seed yield loss, monitoring the pollen quality has become an important risk management tool. In this study, pollen quality analysis methods were evaluated for their suitability for routine quality control of cryopreserved pollen batches. The assessments, including pollen viability, pollen germinability and pollen vigor analysis, were conducted in two locations on a diverse set of cryopreserved tomato and pepper pollen batches. While the viability obtained by Impedance Flow Cytometry (IFC) can be interpreted as the pollen’s potential to germinate, the in vitro germination assay directly quantifies this functionality under given assay conditions. A linear correlation was found between pollen viability obtained by IFC and in vitro germinability. In conclusion, IFC is the most suitable tool for applications and industries requiring a high degree of automation, throughput, repeatability, and reproducibility. In vitro germination assays are suitable for studies within certain temporal and geographic limitations, due to difficulties in standardization. On the other hand, vigor assessments are not sufficiently addressing the needs of the industry due to poor reproducibility and low throughput.

## Introduction

Pollen grains need to achieve highly specialized functions during their life span, such as reaching a stigma after being released from the stamens, germinating on the stigmatic surface, developing a pollen tube that is able to reach the base of the style and enter the ovary, and finally locating and entering the ovule to deliver sperm to a receptive egg and central cell^1,2^. Viable pollen is one of the essential elements for successful plant reproduction, and a prerequisite for the formation of seeds.

The ability to store viable pollen for long periods creates flexibility for breeders and seed producers in agriculture, forestry and horticulture, as it allows for wide hybridization across geographical and seasonal limitations, and thus reduces the coordination required to synchronize flowering and pollen availability for use in pollination^3,4^. In addition, pollen cryopreservation allows to conserve germplasm in a minimum of space, even of rare and endangered species^5^, and can be made available upon request to apply the most optimal pollen load onto the stigma^6^. Successful cryopreservation of pollen has been reported for a diverse group of species, including members of the *Solanaceae* family^7^. Within the *Solanaceae* family, the *Solanum* and *Capsicum* genera are of worldwide importance for human civilization as food source, including cultivated tomato (*Solanum lycopersicum*) and pepper (*Capsicum annuum*) crop plants as two of the most economically important members^8^. *Solanaceae* pollen cryopreservation therefore is a common practice applied by hybrid seed producers worldwide and has particular importance in strategies of securing intellectual property by centralizing pollen production, reducing the female-to-male ratio in multi-year seed production schemes, and pollen production risk management.

One essential parameter of any cryopreservation strategy is the moisture content of the pollen grains, since water balance is crucial for maintaining their viability in time^9,10^. Pollen can be dispersed with different hydration status^11,12^, which is linked to carbohydrate content, physicochemical and ecological features^13^. Pollen that withstands dehydration is termed orthodox. Since the water content of this pollen is less than 30% upon pollen dispersal, these pollen grains are classified as partially dehydrated^11–13^. Partially dehydrated pollen has furrows—axially elongated apertures of high compliance and intricate sculpturing—to allow harmomegathic changes in volume during dehydration and rehydration^13,14^. During dehydration, the pollen wall folds in on itself to prevent further desiccation. The structural patterning of the pollen wall furrows is critical for predictable and reversible folding patterns^15^. Pollen of both pepper and tomato contain furrows and can be considered orthodox pollen^16–18^.

The process of cryopreservation, typically consisting of a sequence of dehydration, cooling, storing, thawing and rehydration, can severely impact the pollen quality, especially if process conditions are not optimal^19^. Measuring the pollen quality along the pollen supply chain is a means of ensuring sufficient pollen quality at the time point of pollination. Therefore, the industry has adopted pollen viability tests as a risk-management tool applied to prevent yield losses due to insufficient pollination success. For the assessment of pollen viability, a wide range of methods have been applied. The majority of such in vitro tests is based on microscopy and determines pollen quality either by measuring the pollen size variation^20^, by detecting metabolic activity or membrane integrity using fluorescent or nonfluorescent dyes^21^, or by quantifying the fraction of pollen grains capable of germination in vitro^22^. Pollen vigor, a measure of the pollen tube growth dynamics, is usually also assessed by microscopy. Microscopic techniques have successfully been used for decades. Data analysis in such assays is typically done by manually quantifying cell subpopulations, or using image processing software tools^23^. More recently, particle counters and flow cytometric methods, such as Coulter Counter and Impedance Flow Cytometry (IFC), have been introduced to this field. As an example, the Ampha Z32 Impedance Flow Cytometer from Amphasys (Switzerland) is a device specifically developed for pollen analysis and is based on the measurement of the electrical properties of individual pollen grains measured in a microfluidic chip^24–26^. All these techniques have advantages and shortcomings when comparing attributes, such as labor intensity, analysis and hands-on time, reliability, potential for standardization, and costs.

The aim of this study was to evaluate and compare pollen analysis methods which are widely applied in hybrid seed production and to identify the most suitable tools to serve the needs of this industry. The technical requirements for such a tool would be that it is simple to operate with a high degree of automation, in order to be used with minimum training. The analysis method should be standardizable and reproducible, to be able to compare results obtained from different geographical sites or from past trials. In addition, the method should have a high throughput to enable processing several thousands of samples per year. Finally, the method must be applicable for the analysis of pollen samples throughout the entire pollen supply chain, and particularly of cryopreserved pollen batches. This latter aspect was defined as a focus topic of this work, because, to the best of our knowledge, a thorough characterization and comparison of pollen analysis methods using cryopreserved pollen has not been published yet and would not only be highly relevant for the hybrid seed production and breeding industry, but also for other fields of plant reproduction.

In this study, a diverse set of cryopreserved tomato and pepper pollen samples, originating from multiple geographical locations and production years, was characterized by two independent laboratories in the Netherlands and in Switzerland, by different operators and using three widely used analysis methods: Pollen viability using IFC, in vitro pollen germinability, and pollen vigor. This setup allowed us to assess the potential of each method for the seed industry.

## Results

### Correlation between IFC data and microscopy

**Figure 1:**
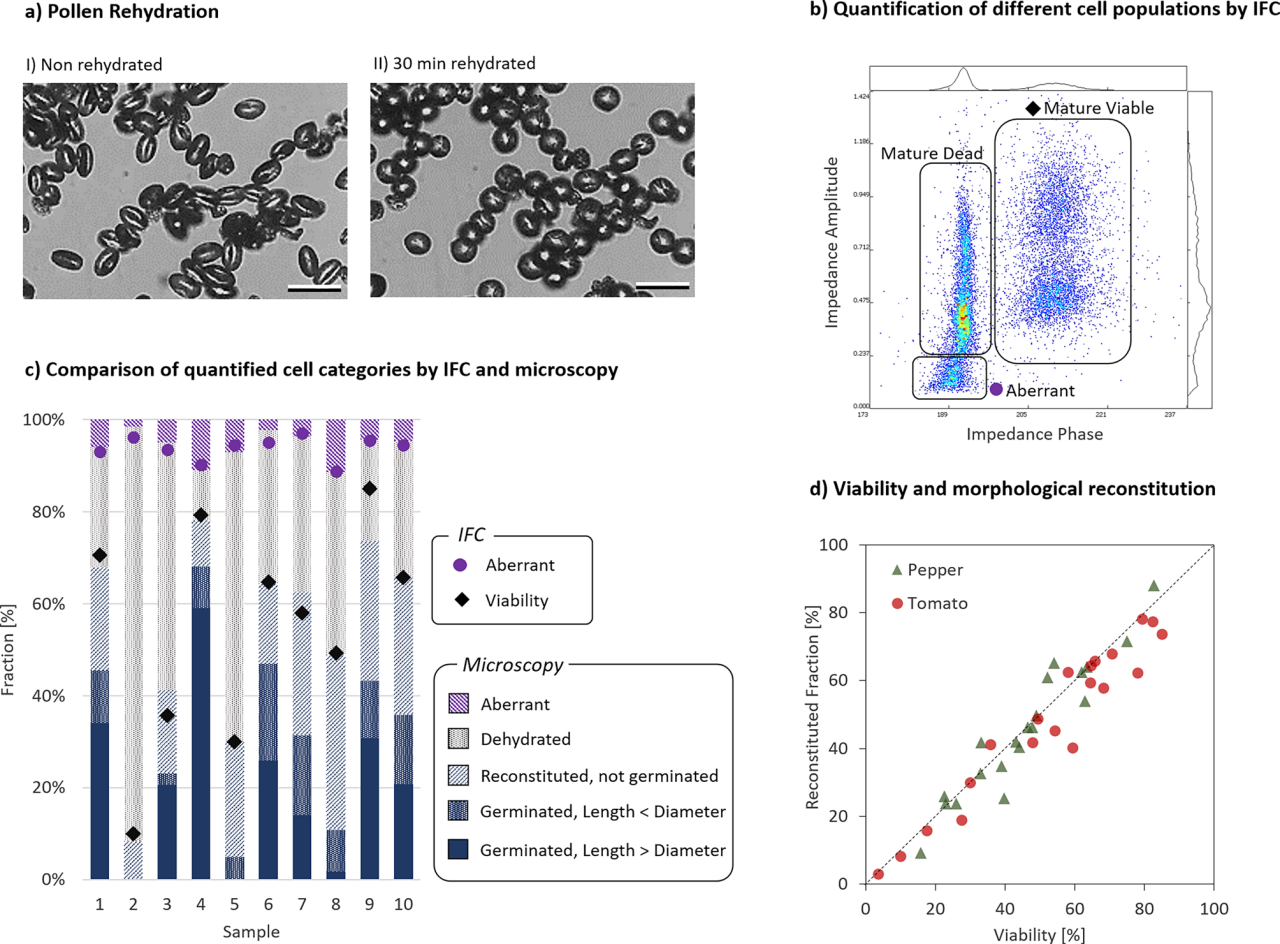
(**a**) Morphological differences of a cryopreserved tomato pollen sample (I) and the same sample after 30 min of rehydration at 95–99% relative humidity (II). During the rehydration process, cells reconstitute from a furrowed to a more spherical morphology (scale bar = 50 μm). (**b**) Typical IFC data gating strategy for the analysis of *Solanaceae* pollen. Gatings are used to quantify viable, dead, and aberrant pollen grains in Phase—Amplitude scatterplots. Note that each dot corresponds to one measured cell. (**c**) Quantification of individual tomato pollen cell populations by IFC and microscopy. Bars: Classification based on microscopy. Points: Classification by IFC (**d**) Correlation between IFC viability and the reconstituted pollen fraction for pepper and tomato pollen samples. Note that the reconstituted fraction shown here also includes germinated cells.

In the first step of the analysis, microscopic data and IFC data from the same samples were compared. In the microscopic images of the in vitro pollen germination assays, distinct morphological subtypes of pollen grains were identified (Table 1) and quantified. The relative quantities are shown as stacked bars in Fig. 1c.

**Table 1:** Categorization of pollen grains into different morphological subtypes.

Category	Description	Example image
Aberrant	Aberrant grains have been associated with abortion of the microspore development, e.g. due to heat stress^29,30^	
Dehydrated	Pollen failing to morphologically reconstitute after rehydration and suspension in the germination buffer. Furrows can clearly be identified, resembling a “coffee bean”. See Fig. 1a–I	
Reconstituted, not germinated	This category comprises pollen grains which were morphologically reconstituted by rehydration and suspension in germination buffer but did not form a pollen tube. See Fig. 1a–II	
Reconstituted, pollen tube emerging	These pollen grains formed a pollen tube, but the tube length was shorter than the pollen’s diameter	
Reconstituted, pollen tube longer than pollen diameter	These pollen grains formed pollen tubes with tubes longer than the pollen diameter

While morphological characteristics are used to classify cells in microscopy, the electrical properties of cells are measured with IFC. IFC data analysis is based on gating of 2-dimensional phase-amplitude scatter plots, and typically involves quantification of viable, dead, and aberrant cells. A representative example of such analysis is shown in Fig. 1b. The viable and aberrant cell fractions obtained from IFC were added to the bars in Fig. 1c as data points.

The comparison between IFC and microscopy data showed that:

1. Cryopreserved pollen batches can have very variable compositions of individual cell subpopulations (Fig. 1c).
2. The quality of cryopreserved pollen batches can vary substantially (Fig. 1c), posing a risk of insufficient pollination when using low quality batches, and providing an opportunity of diluting high quality batches with diluents (e.g. *Lycopodium* spores).
3. Viable pollen identified by IFC corresponded to the pollen fraction which could morphologically reconstitute after cryopreservation (Fig. 1d).
4. Both techniques quantified aberrant cells similarly (Fig. 1c).
5. The fraction of pollen which was viable but failed to germinate under the given germination assay conditions was highly sample-specific and ranged from 8.7 to 43.3%. Such cells managed to reconstitute morphologically, but not functionally (Fig. 1c).

### Comparing results of different pollen quality test methods

**Figure 2:**
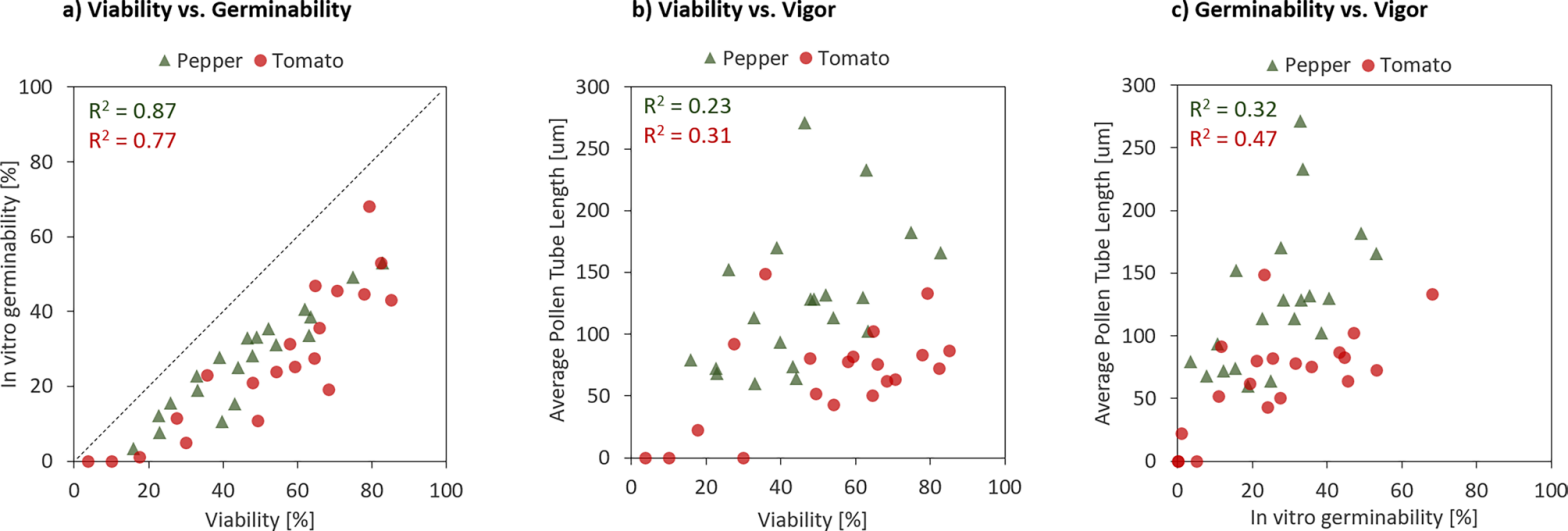
Comparison of pollen quality analysis using IFC viability, in vitro germination and vigor assessment by determining the average pollen tube length. Each datapoint corresponds to a measurement result of either pepper (green) or tomato (red). (**a**) Comparison of pollen viability with in vitro germination. (**b**) Comparison of pollen viability with the average pollen tube length. (**c**) Comparison of the in vitro germination with the average pollen tube length.

Comparison of the results obtained from all 20 samples at both test sites in the Netherlands and Switzerland indicated a strong positive correlation between IFC viability and in vitro germinability (Fig. 2a , R^2^= 0.77 for tomato, R^2^ = 0.87 for pepper). The in vitro germinabilities were on average 26% and 19% lower than the IFC viabilities for tomato and pepper, respectively. A positive trend between viability and pollen tube length was found as well, indicating that pollen batches of a higher quality tend to contain more vigorous pollen, forming longer pollen tubes (Fig. 2b). A similar observation was made when comparing in vitro germinability with pollen tube length (Fig. 2c).

### Reproducibility of different test methods

**Figure 3:**
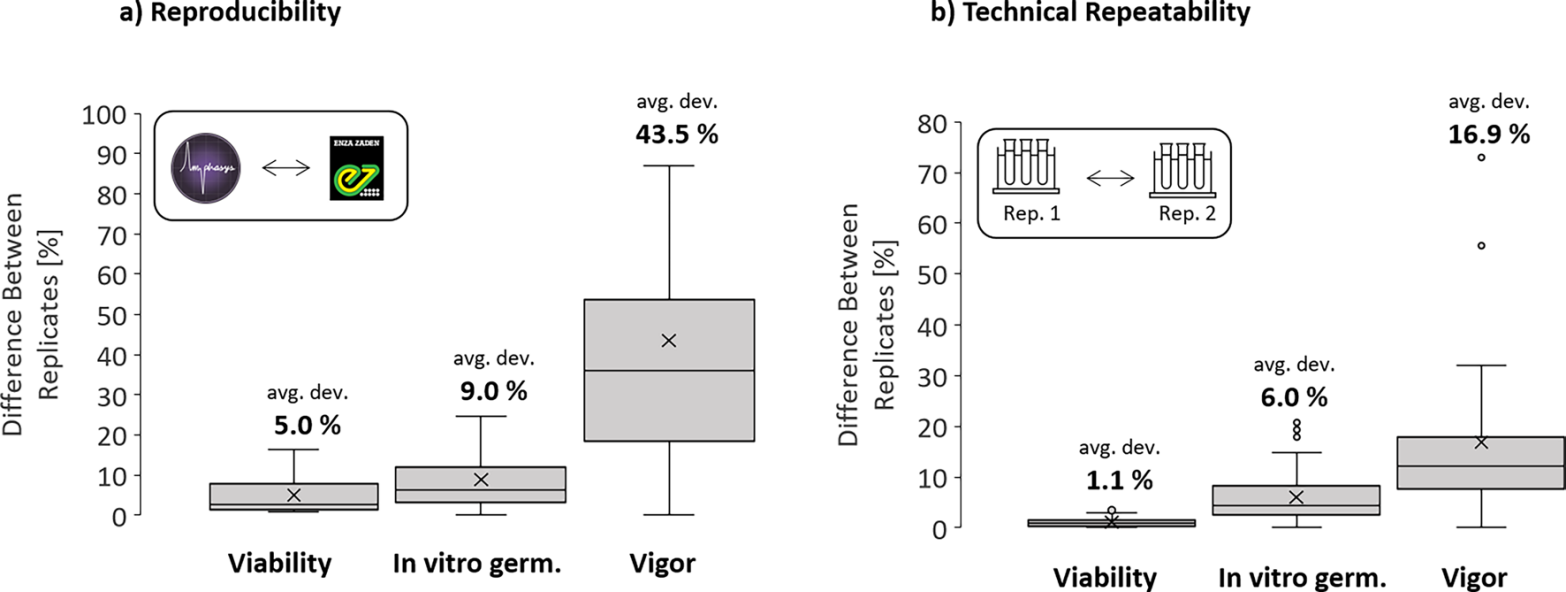
Reproducibility and repeatability of test results by different test methods. (**a**) Assessing the reproducibility of test results by performing tests in different locations by different operators. (**b**) Assessing the technical repeatability by performing the same assay twice on the same day by the same operator, conducted at both locations.

As the same set of samples was available at two geographical locations and the test setup was standardized, the reproducibility of the test methods could be compared. The IFC viability results obtained in the Netherlands and Switzerland correlated very well, with an average deviation of 5% (Fig. 3a). The results obtained by in vitro germinability were slightly less reproducible with an average deviation of 9%, and the vigor assessment resulted in large differences.

The technical repeatability was assessed by comparing the test results obtained from two technical replicates. The results in Fig. 3b indicate a very high repeatability for IFC viability, a moderate repeatability for in vitro germination and a poor repeatability for the vigor assessment.

## Discussion

A solid link between morphologically distinct cell populations and IFC datapoint clusters has been established for tomato and pepper pollen (Fig. 1b,c). Viable cells identified by IFC correspond to morphologically reconstituted cells, which have a round and large appearance (Table 1 and Fig. 1d). Only such viable, well hydrated cells are able to germinate. As shown in Fig. 2a, a fraction of morphologically reconstituted cells, however, fails to reconstitute to a functional level. This may be attributed to heterogeneity in the pollen maturity. Due to asynchronies during pollen grain development, there may be differences in the metabolic state of single pollen grains upon anther opening^27^. Carbohydrate content of mature pollen can be one of the variables resulting in dissimilar physiological reactions of single pollen grains, like their ability to control desiccation, to (re)hydrate and to germinate. Furthermore, it is also conceivable that a loss of germination capacity results from irreversible damage due to pollen processing, such as dehydration, cryopreservation and thawing.

**Figure 4:**
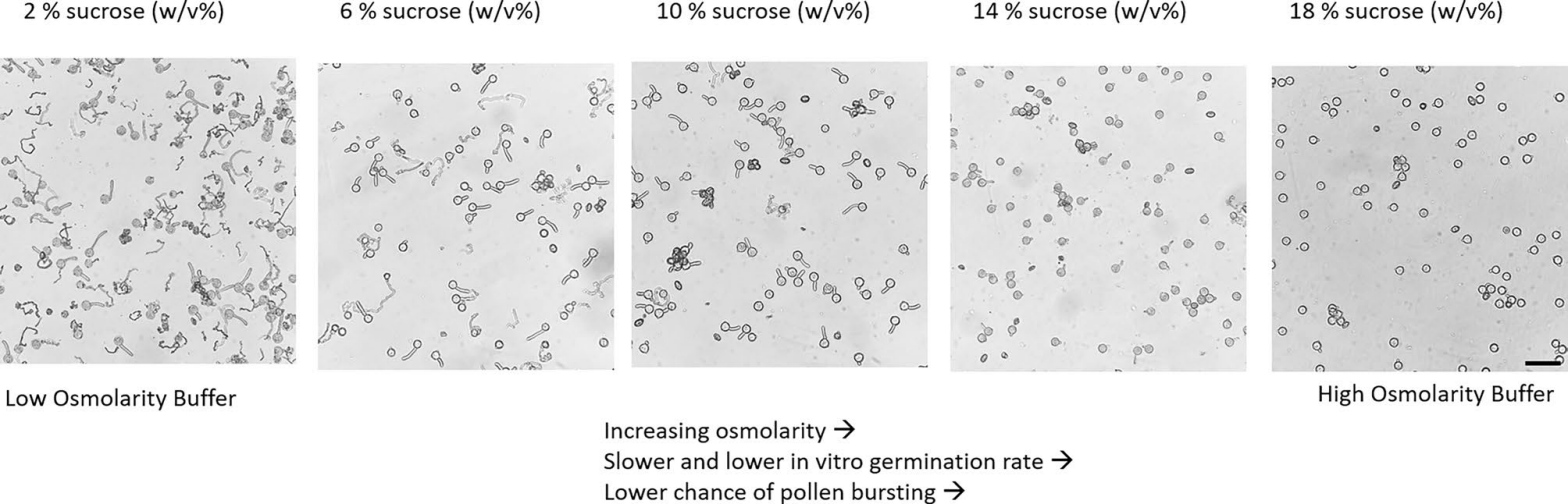
Effect of germination medium osmolarity on the in vitro tomato pollen germination rate and pollen integrity. High osmolarity buffers reduce the germination rate and the risk of pollen bursting (scale bar = 100 μm).

Another aspect to consider is the strong effect of the assay conditions on the outcome of the in vitro germination, i.e. the fraction of viable but non-germinating cells strongly depends on the buffer composition. High osmolarity buffers reduce the germination rate and the risk of pollen bursting due to differences in the osmotic potential, leading to limited water uptake of the pollen grains (Fig. 4)^28^. Therefore, an optimized buffer, resulting in the highest possible in vitro germination capacity, with low pollen bursting rate, should be used in every pollen germination assay. In addition, buffer osmolarity seems to be directly linked with pollen vigor, due to its effect on pollen germination speed.

Besides mature viable cells, mature dead cells were also quantified in this set of experiments. Mature dead cells maintain their furrowed morphology during rehydration and suspension in liquid media. These cells lost their capability for reconstitution and do never germinate. Due to their distinct morphology and dielectric properties, they can easily be identified using microscopy and IFC (Table 1 and Fig. 1b).

Aberrant cells are a category of cells characterized by a small amorphous morphology. These cells have been described as aborted immature cells^29,30^. They are again easily identifiable with microscopy and IFC, and can be indicative of problems during the microspore development process, e.g. resulting from heat-stress during its susceptible developmental phase.

The linear correlation between results obtained with IFC and microscopy shown in Figs. 1d and 2a is remarkable given the very different measurement principles applied in both methods. While morphological features were used to classify cells in microscopy, IFC measures the dielectric properties of the cells in the sample. The findings indicate that IFC viability can be interpreted as the pollen fecundation competence, while the in vitro germination assay directly quantifies this functionality under the given assay conditions.

The linear correlation between pollen vigor and both germinability and viability was generally rather low. This was to some extent expected given the diverse pool of samples used in this study, coming from several different production sites across the globe, being stored for variable durations, and each representing a mixture of multiple pollen collections. The tedious measurement of the length of individual pollen tubes and the strong dependency of the tube growth dynamics on buffer composition and temperature prove that the vigor assessment does not fulfil the requirements for routine quality control of cryopreserved pollen batches.

The experimental design of this study (same pollen samples, different sites, different operators, different timepoints) further allowed to evaluate the performance of the different test methods applied. IFC measurements showed a remarkably high reproducibility (5% difference) and technical repeatability (1.1% difference) for both tomato and pepper. These factors were acceptable for the in vitro germination assay (9% and 6% respectively), while the vigor assessment showed a poor reproducibility (43.5%) and repeatability (16.9%), indicating the difficulty of standardizing the test setup. One factor directly influencing the reproducibility and repeatability is the sample size. While several hundreds of cells are usually quantified in microscopic tests with manual classification of subpopulations, 10′000 cells can easily be assessed using IFC within a few seconds, leading to a higher statistical precision. One practical implication of such high technical repeatability is the higher capacity for biological replication. This is particularly important for the analysis of fresh unprocessed pollen from individual flowers or stamens, as the variation observed in such samples can be substantial.

In conclusion, both in vitro germination and IFC viability are suitable methods for the quality analysis of cryopreserved tomato and pepper pollen. The results obtained with both methods are linearly correlated. Importantly, following a standardized protocol is strongly recommended for the germination assay. This includes optimization of the buffer recipe, controlled assay temperature and duration, and clear specifications of how to classify different pollen morphologies and germination levels. The pollen viability assessment using IFC, on the other hand, is an inherently standardized, high-throughput method which can be easily implemented across locations and users. Limitations of the IFC viability method include higher investment costs, due to the need to purchase a pollen analyzer and related accessories, in addition to hands-on training before use. While the in vitro germination assay can be a suitable method to address specific questions limited in resources, or if pollen quality is not investigated on a routine basis (e.g. a short trial conducted in one research site), IFC is recommended for industries and academia working on plant reproduction projects with a wider scope, involving multiple operators, sites and requiring comparability of data across a larger time window.

## Materials & methods

### Sample source and distribution

Pollen donor plants (Enza Zaden, The Netherlands) were cultured in net houses or greenhouses under local standard conditions. Pepper (*Capsicum annuum*) plants were cultivated in Chile, Tanzania and the Netherlands in 2018–2020. Tomato (*Solanum lycopersicum*) plants were cultivated in Chile, Peru, China, Spain, Tanzania and the Netherlands in 2014–2019. Each pollen sample used in this study is obtained from a minimum of 50 plants from one male parental genotype, cultivated in one specific year on one of the locations mentioned. Pollen was collected from donor plants with permission, as covered by a Seed Production Contract between the plant grower and Enza Zaden. Pollen collection included the harvest of pollen either directly from open flowers, or indirectly after drying of the anthers. Pollen was subsequently dried according to the individual plant grower’s standards, placed in a − 20 °C freezer, and shipped in cryopreserved condition (below 0 °C) to the Netherlands. Pollen was stored in a − 80 °C freezer in the Netherlands for several years and transferred to a − 20 °C freezer in 2021. Pollen replicate subsamples were taken and shipped in cryopreserved conditions to Switzerland within 24 h, after which they were stored in a − 20 °C freezer as well.

### Sample preparation

Creation of stock samples: Ten cryopreserved samples of pepper and ten of tomato were thawed (15 min, 20–25 °C), after which a small aliquot was rehydrated in a high-humidity box, ensuring saturation (95–99% RH, 45 min, 20–25 °C). Rehydrated pollen was suspended in 1 ml AF6 IFC measurement buffer (Amphasys AG, Switzerland), or 1 ml germination buffer, and filtered through a 50 µm CellTrics filter (Sysmex, Switzerland) into a clean Eppendorf tube. For pepper, the germination buffer had the following composition: 2% sucrose (w/v%), 24% PEG 3000 (w/v%), 1.62 mM H_3_BO_3_, 1.3 mM Ca(NO_3_)_2_, 0.81 mM MgSO_4_, 0.99 mM KNO_3_. For tomato, the germination buffer recipe from Müller et al., 2016 was used^31^.

Sample preparation specific for IFC: From each sample, two aliquots of 300 μl of each pollen stock sample were transferred into clean Eppendorf tubes, generating two technical replicates. Then, 1 ml AF6 was added to each tube. Samples were briefly mixed, and then incubated for 15 min (tomato) or 10 min (pepper) at room temperature. After the incubation, samples were homogenized by carefully inverting the tubes, and then analyzed for pollen viability.

Sample preparation specific for in vitro pollen germination: Wells of a 12-well Cellstar cell culture plate (Greiner Bio-One, Switzerland) were filled with 1 ml germination buffer. Then, 100 μl of each pollen stock sample was transferred into individual wells. The samples were mixed by gently moving the plate. Then, the samples were incubated at 25 °C for 75 min (tomato) or 3 h (pepper), before analysis by microscopy.

### Pollen viability measurement

Pollen viability was measured using an Ampha Z32 Impedance Flow Cytometer (Amphasys AG, Switzerland). For the measurement, a 120 μm chip (Amphasys AG, Switzerland) was used, in combination with the crop-specific standard templates provided by Amphasys. Two technical replicates, both including 10 × 10^3^ cells, were measured for each sample. After the measurement, individual cell subpopulations (mature viable, mature dead and aberrant) were quantified using AmphaSoft 2.1.6 software (Amphasys AG, Switzerland).

### Pollen germination assay and vigor assessment

A minimum of 3 images containing a total minimum of 300 pollen grains per sample were classified using brightfield microscopy at 100 × magnification (Leica DM IRB and Leica DMi8). Aberrant, dehydrated, morphologically reconstituted, and germinated cells (pollen tube length < cell diameter and pollen tube length > cell diameter) were quantified using the Fiji Cell Counter Plug-in. Examples are shown in Table 1. For the pollen vigor assessment, the same microscopic images acquired for germination were used to measure the pollen tube length of germinated pollen grains, reached after the defined incubation time period, using the Arrow Tool function in Fiji. The length of individual pollen tubes was measured in pixels and converted to µm using the microscopic scale bar. For each sample the mean pollen tube length was determined and used for data visualization.

All experiments were conducted at two different sites (Amphasys, Switzerland; Enza Zaden, The Netherlands) by different operators and at different time points, using subsamples of the same pollen batch. All methods were carried out in accordance with relevant guidelines and regulations.

## Data Availability

The datasets generated and analyzed during the current study are available from the corresponding author on reasonable request.
